# Supplementary material for: Fam102a translocates Runx2 and Rbpjl to facilitate Osterix expression and bone formation
Source: Nat Commun. 2025 Jan 2;16:9. doi: 10.1038/s41467-024-55451-z (PMC11695619; doi:10.1038/s41467-024-55451-z)
Supplement: Supplementary file 1 — Supplementary Information [file 41467_2024_55451_MOESM1_ESM.pdf]

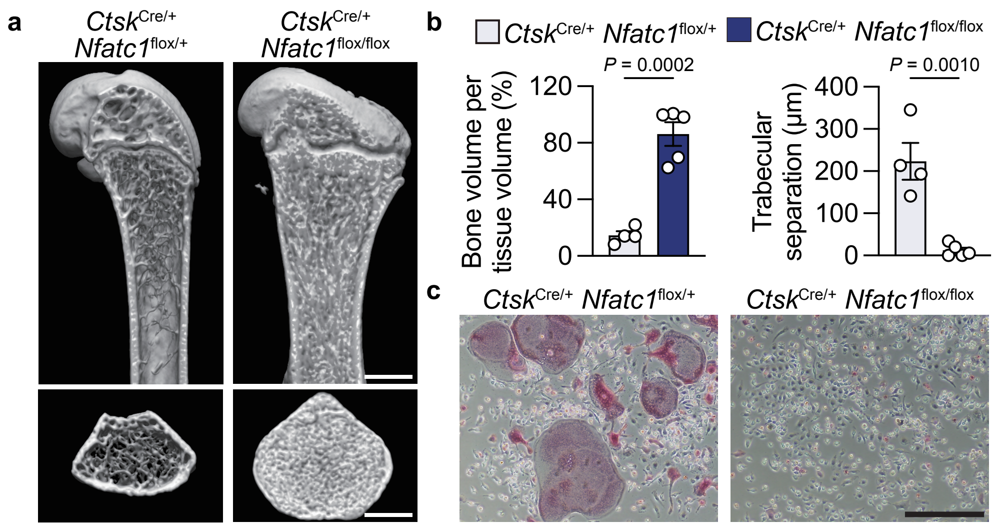

Supplementary Fig. 1: Mice with conditional deletion of *Nfatc1* in osteoclasts exhibit a severe osteopetrotic phenotype.

a, Representative μCT images of the distal femur in 3-week-old male *Ctsk*$^{Cre/+}$ *Nfatc1*$^{flox/+}$ and *Ctsk*$^{Cre/+}$ *Nfatc1*$^{flox/flox}$ mice (scale bar, 1 mm). **b,** μCT analysis of the distal femur in 3-week-old male *Ctsk*$^{Cre/+}$ *Nfatc1*$^{flox/+}$ and *Ctsk*$^{Cre/+}$ *Nfatc1*$^{flox/flox}$ mice (n = 4–5). **c,** *In vitro* osteoclast differentiation of *Ctsk*$^{Cre/+}$ *Nfatc1*$^{flox/+}$ and *Ctsk*$^{Cre/+}$ *Nfatc1*$^{flox/flox}$ splenocytes (scale bar, 0.4 mm). Three independent experiments were performed. Data are shown as the mean ± SEM. Statistical analyses were performed using unpaired two-sided Student' s *t* test. Source data are provided as a Source Data file.

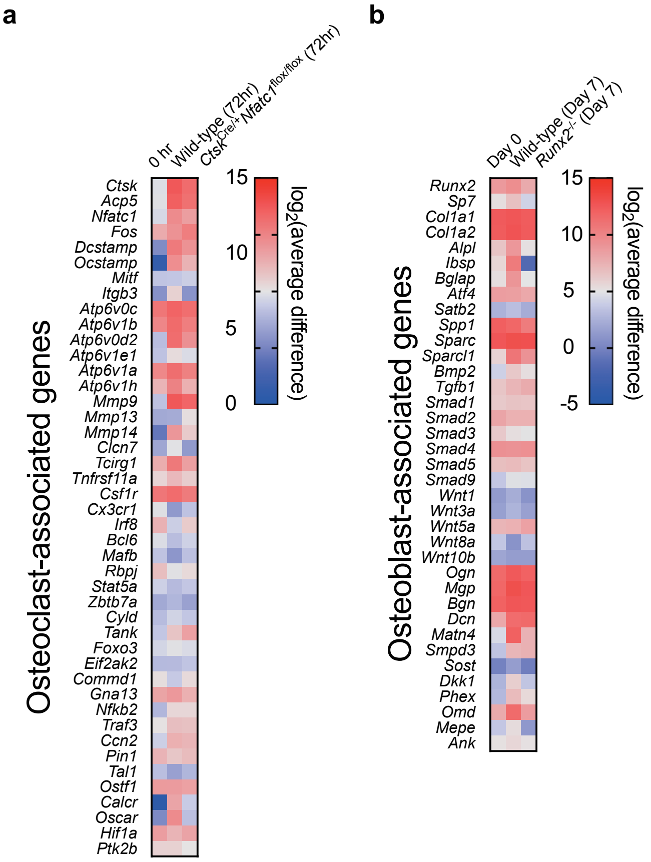

Supplementary Fig. 2: mRNA expression of the genes asscociated osteoclast or osteoblasts.

a,b, The expression of genes associated with osteoclasts or osteoblasts in *Nfatc1*–deficient osteoclasts (**a**) and *Runx2*–deficient osteoblasts (**b**), respectively, were visualized by heatmap. Source data are provided as a Source Data file.

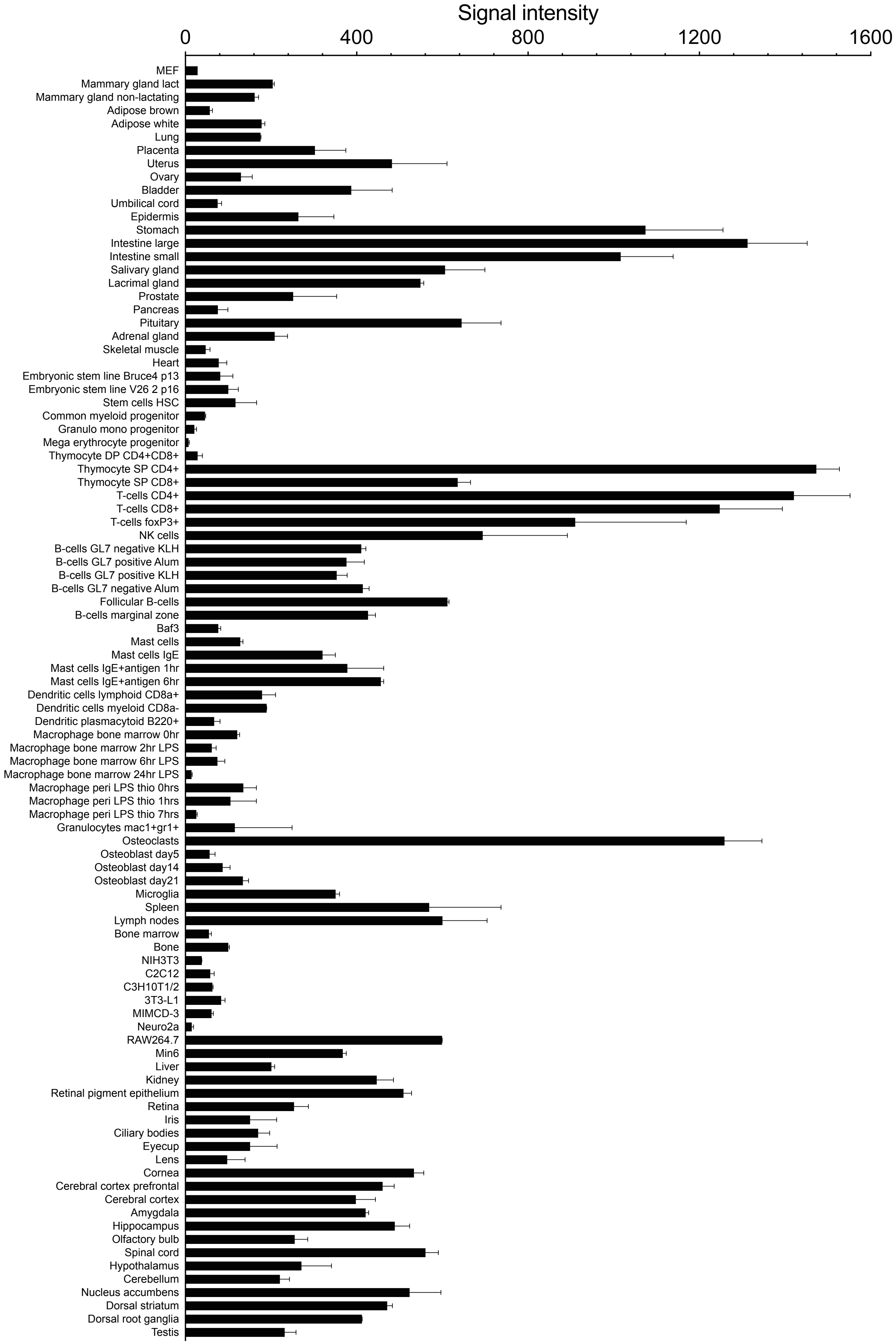

Supplementary Fig. 3: *Fam102a expression* in various mouse tissues and cells.

Expression profile of *Fam102a* among mouse tissues and cells (data from BioGPS, http://biogps.org/). Each bar represents the normalized intensity of the amount of *Fam102a* mRNA in each tissue and cell. Source data are provided as a Source Data file.

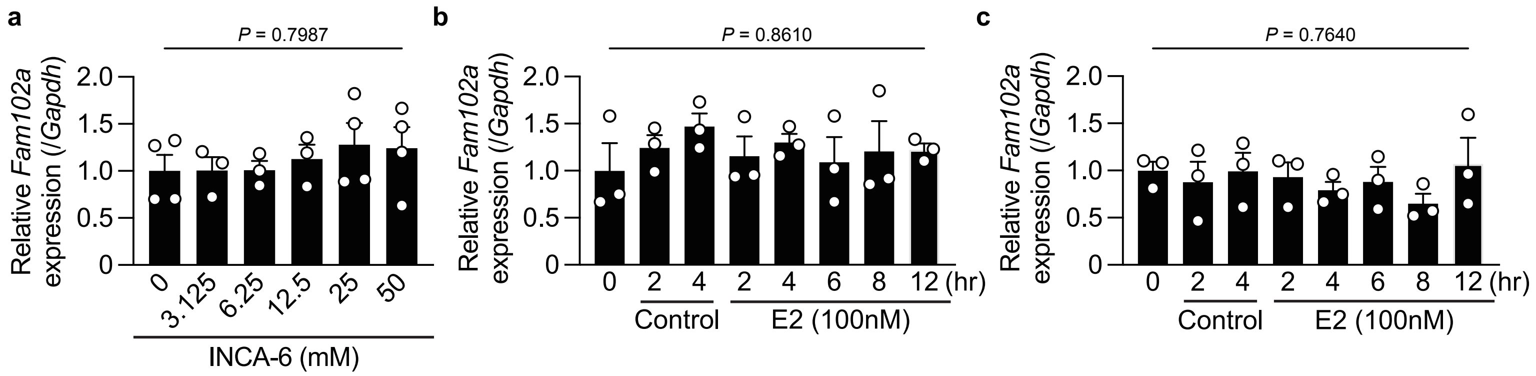

Supplementary Fig. 4: *Fam102a* is expressed independently of estrogen signal in osteoblasts and osteoclasts.

a, *Fam102a* mRNA expression in osteoblastic MC3T3-E1 cells in response to INCA-6, which inhibits NFAT activation (n = 3 or 4; independent experimental replicates). **b,c,** *Fam102a* mRNA expression in osteoblastic MC3T3-E1 cells (**b**) and primary osteoclast precursor cells (**c**) in response to 17β-estradiol (E2) or Ethanol (Control) treatment (n = 3; independent experimental replicates). Data are shown as the mean ± SEM. Statistical analyses were performed using one-way ANOVA and Tukey’ s *post hoc* test. Source data are provided as a Source Data file.

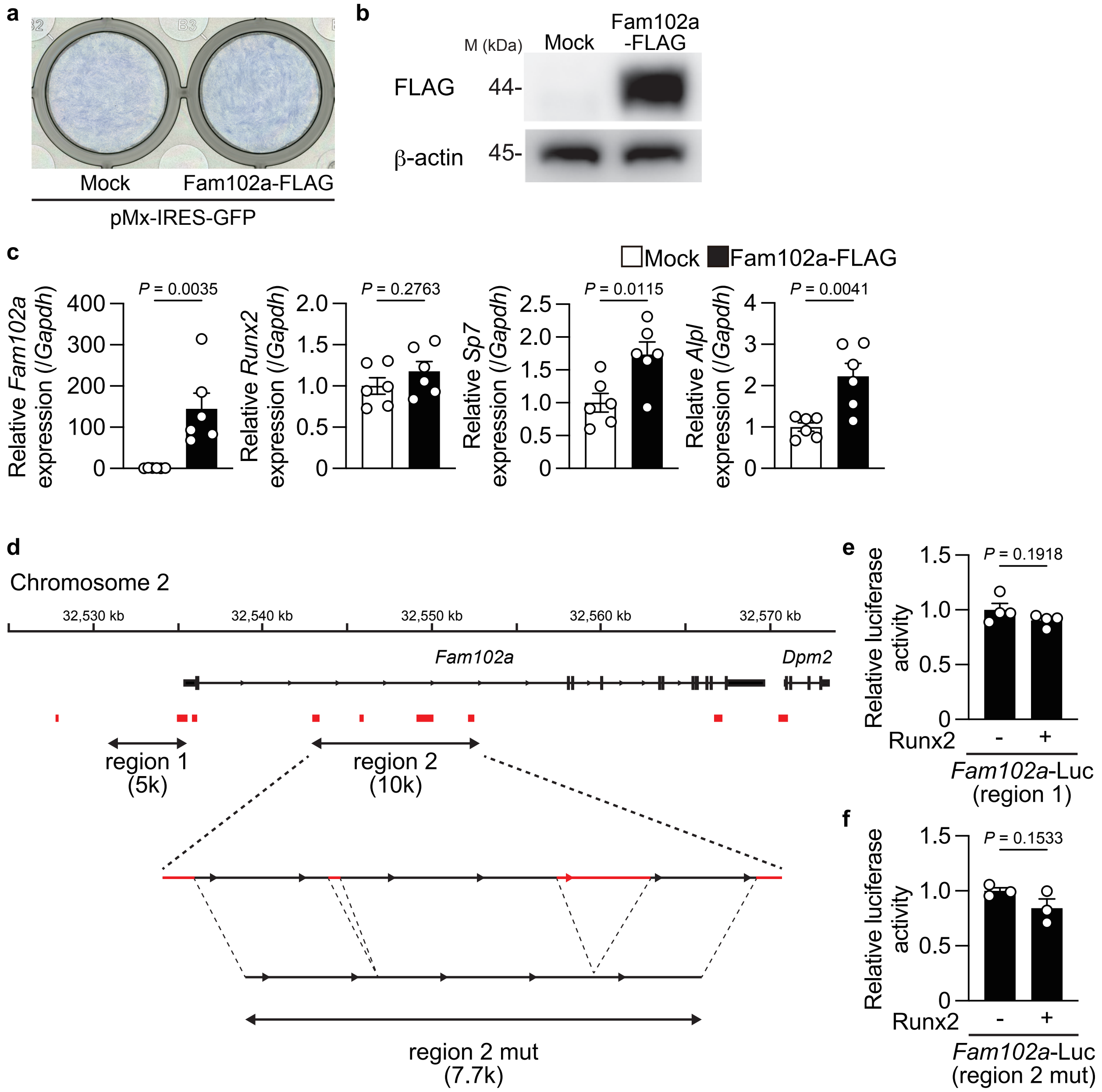

Supplementary Fig. 5: Overexpression of Fam102a promoted osteoblast differentiation.

a–c, The effect of retroviral overexpression of Fam102a on ALP staining (a), Fam102a protein expression (b), and osteoblastic gene expressions (c) in MC3T3-E1 cells cultured in osteogenic medium for 7 days (n = 6; independent experimental replicates). **d,** Schematic view of Runx2-binding sites (red bars) in mouse *Fam102a* promotor and enhancer region visualized using ChIP-Atlas. Lower section of the figure, targeting strategy to generate mutated region 2. **e,** The effect of Runx2 expression on the reporter activity of *Fam102a*-Luc region 1 (**e,** n = 4; independent experimental replicates) and region 2 mut (**f,** n = 3; independent experimental replicates) in MC3T3-E1 cells. M, molecular mass. Data are shown as the mean ± SEM. Statistical analyses were performed using Student’ s t test. Source data are provided as a Source Data file.

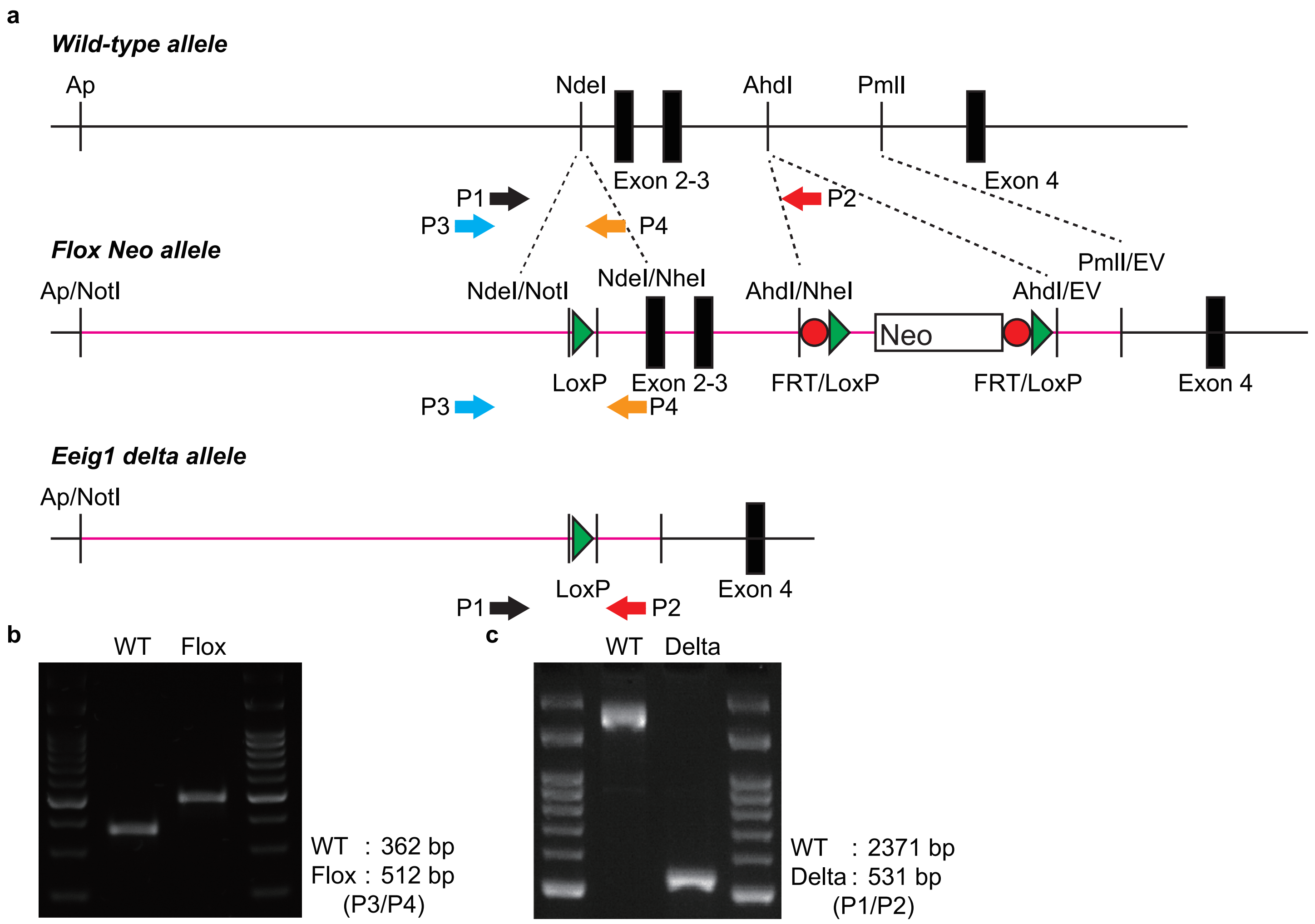

Supplementary Fig. 6: Generation of *Fam102a* floxed and deleted mice.

a, Targeting strategy to generate *Fam102a* floxed and deleted mice. The arrows below the diagram of the wild-type allele indicate the positions of the primers (P1, P2, P3 and P4) used for PCR genotyping. **b,** Genotyping of floxed allele of *Fam102a* by PCR using P3 and P4 primers. **c,** Genotyping of deleted allele of *Fam102a* by PCR using P1 and P2 primers. WT, wild-type.

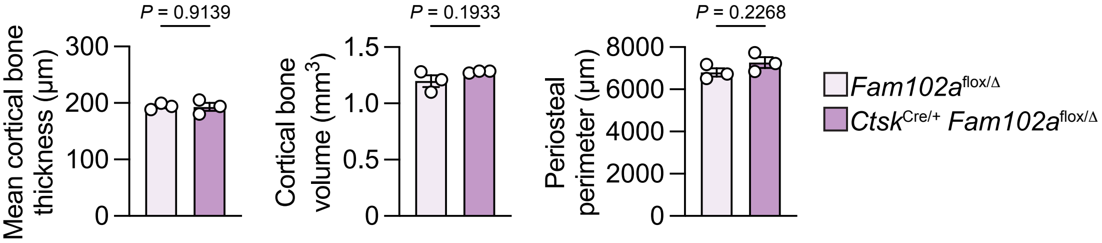

Supplementary Fig. 7: The effect of *Fam102a* deficiency in *Ctsk*^{Cre} positive cells on cortical bone.

Cortical bone analysis as measured by µCT in 12-week-old male *Ctsk*$^{Cre/+}$ *Fam102a*$^{flox/\Delta}$ mice and their littermate controls (n = 3). Data are shown as the mean ± SEM. Statistical analyses were performed using unpaired two-sided Student’ s t test. Source data are provided as a Source Data file.

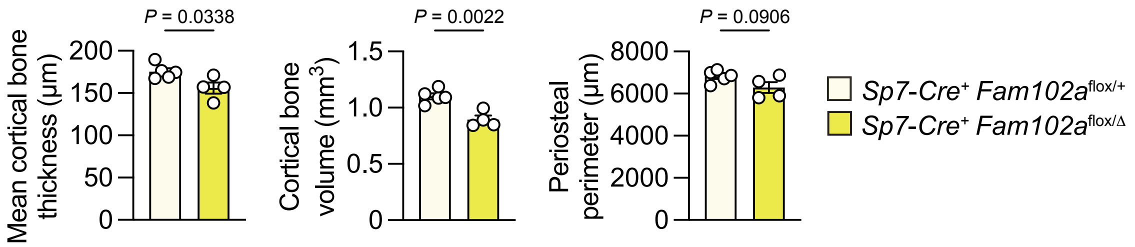

Supplementary Fig. 8: The effect of *Fam102a* deficiency by *Sp7-Cre* on cortical bone.

Cortical bone analysis as measured by μCT in 12-week-old male *Sp7-Cre*$^{+}$ *Fam102a*$^{flox/\Delta}$ mice and their littermate controls (*Sp7-Cre*$^{+}$ *Fam102a*$^{flox/+}$: *n* = 5, *Sp7-Cre*$^{+}$ *Fam102a*$^{flox/\Delta}$: *n* = 4). Data are shown as the mean ± SEM. Statistical analyses were performed using unpaired two-sided Student’ s *t* test. Source data are provided as a Source Data file.

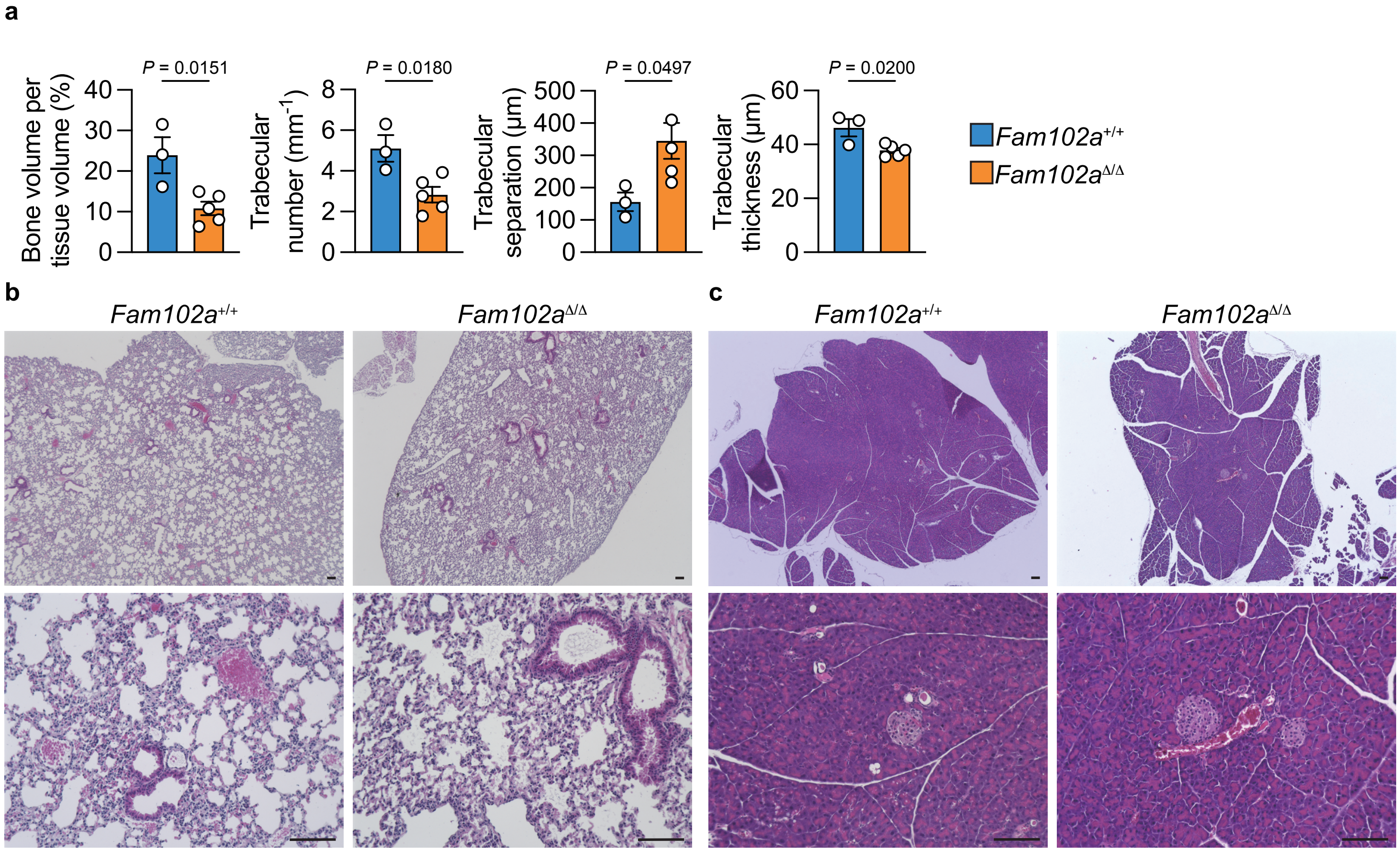

Supplementary Fig. 9: Bone phenotype of female *Fam102a*$^{\Delta/\Delta}$ mice and histological analysis of the lungs and pancreas in *Fam102a*$^{\Delta/\Delta}$ mice.

a, µCT analysis of the distal femur in 12-week-old female *Fam102a*$^{\Delta/\Delta}$ mice and their littermate controls (*Fam102a*$^{+/+}$: *n* = 3, *Fam102a*$^{\Delta/\Delta}$: *n* = 4). **b,c,** Representative images of HE-stained lung (**b**) and pancreas (**c**) in 12-week-old male *Fam102a*$^{\Delta/\Delta}$ mice (upper panel, low magnification (×4); lower panel, high magnification (×20); scale bar, 100 µm). Three independent experiments were performed. Data are shown as the mean ± SEM. Statistical analyses were performed using unpaired two-sided Student’ s *t* test (**a**). Source data are provided as a Source Data file.

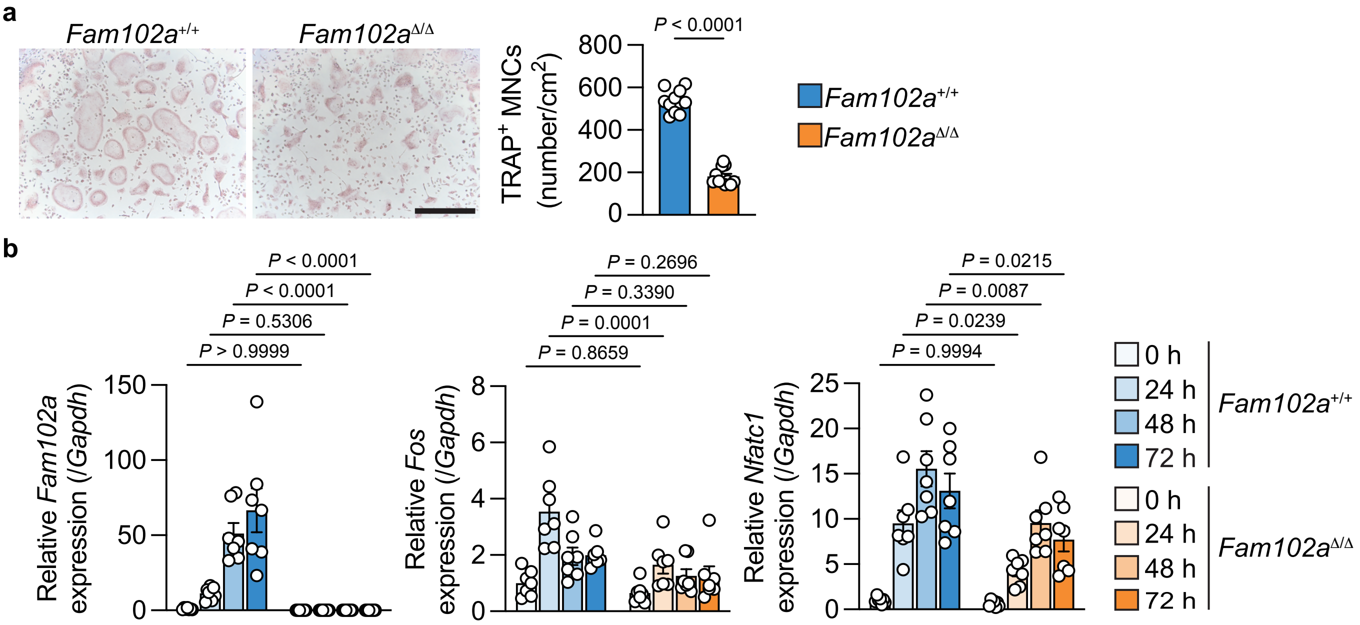

Supplementary Fig. 10: Deficiency of *Fam102a* suppressed osteoclast differentiation.

a, *In vitro* osteoclast differentiation of *Fam102a*$^{+/+}$ and *Fam102a*$^{\Delta/\Delta}$ BMMs shown by TRAP staining (*n* = 10; independent experimental replicates) (scale bar, 0.4 mm). **b,** mRNA expression of osteoclastic genes during differentiation in *Fam102a*$^{+/+}$ and *Fam102a*$^{\Delta/\Delta}$ bone marrow cells (*n* = 7; independent experimental replicates). Data are shown as the mean ± SEM. Statistical analyses were performed using unpaired two-sided Student' s *t* test (**a**) or two-way ANOVA and Sidak' s *post hoc* test (**b**). Source data are provided as a Source Data file.

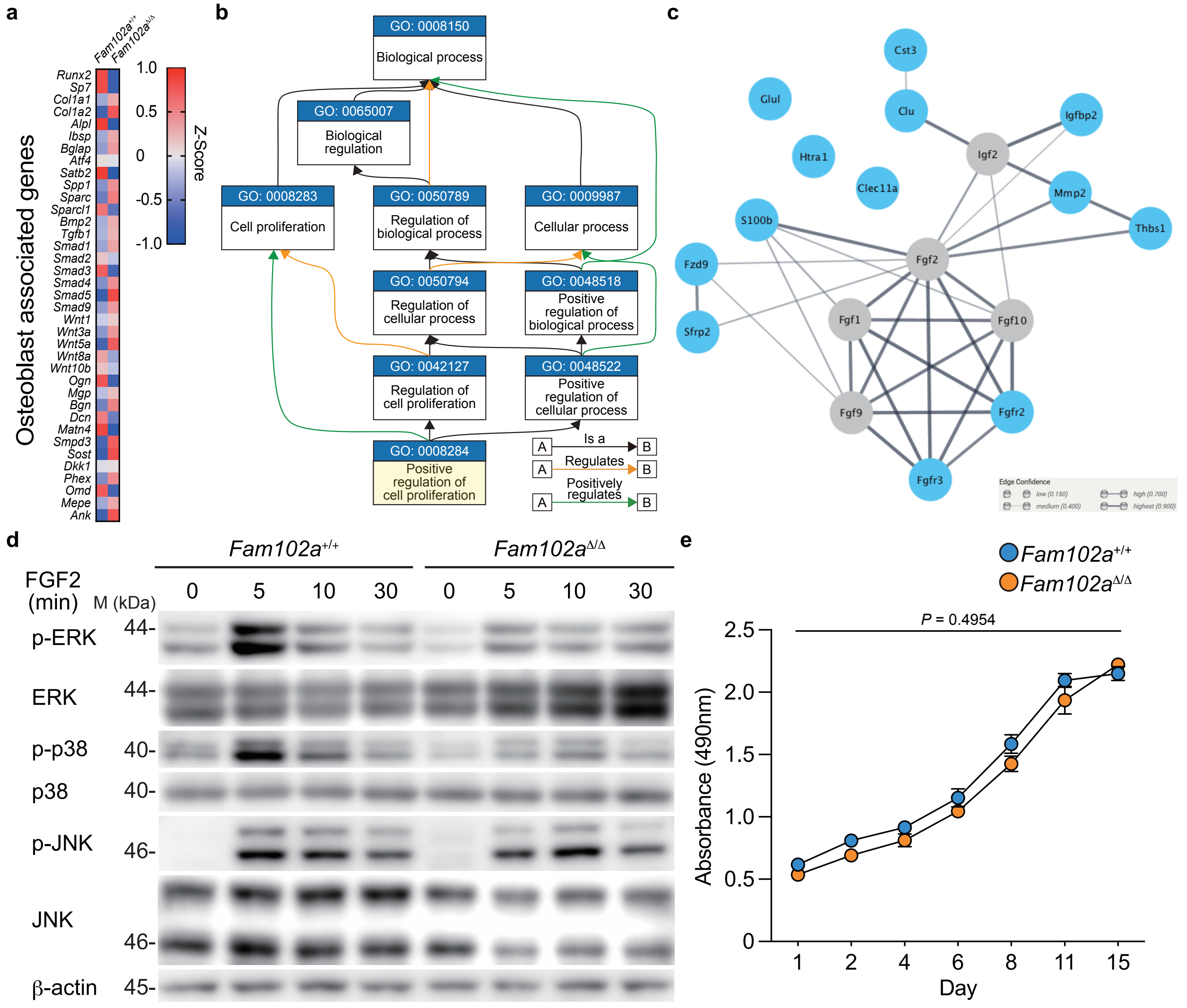

Supplementary Fig. 11: Deficiency of *Fam102a* suppressed FGF2 signaling, whereas cell proliferation was maintained.

a, The expression of genes asscoiated with osteoblast differentiation in *Fam102a*$^{+/+}$ and *Fam102a*$^{\Delta/\Delta}$ calvarial cells, visualized by heatmap. **b,** Gene ontology (Biological process) pathway analysis was performed using QuickGO on genes differentially decreased in *Fam102a*$^{\Delta/\Delta}$ osteoblasts relative to *Fam102a*$^{+/+}$ osteoblasts. **c,** The PPI network was visualized using the STRING database. Light blue circle nodes: 13 differentially expressed genes in Gene ontology terms (positive regulation of cells); gray circle nodes: putative proteins related to the PPI. **d,** FGF2-induced phosphorylation of ERK, p38, and JNK in *Fam102a*$^{+/+}$ and *Fam102a*$^{\Delta/\Delta}$ calvarial cells cultured for 7 days. Three independent experiments were performed. **e,** Cell proliferation in *Fam102a*$^{+/+}$ and *Fam102a*$^{\Delta/\Delta}$ calvarial cells (n = 4–8; independent experimental replicates). M, molecular mass. Data are shown as the mean ± SEM. Statistical analyses were performed using two-way ANOVA and Sidak’ s *post hoc* test (**e**).Source data are provided as a Source Data file.

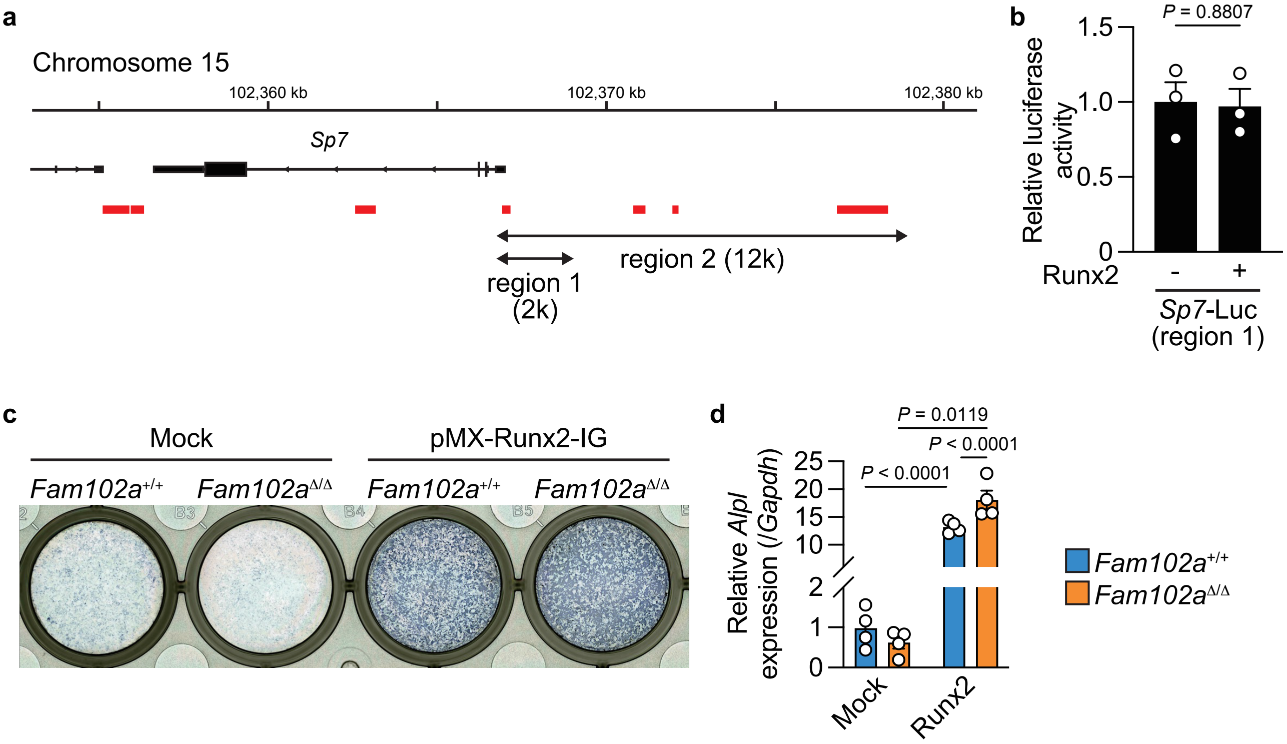

Supplementary Fig. 12: Overexpression of Runx2 in *Fam102a*–deficient osteoblasts rescued impaired osteoblast differentiation.

a, Schematic view of Runx2-binding sites (red bars) in the mouse *Sp7* promoter and enhancer regions visualized using ChIP-Atlas. **b,** The effect of Runx2 expression on the reporter activity of *Sp7*-Luc (region 1) in MC3T3-E1 cells (n = 3; independent experimental replicates). **c**, *In vitro* osteoblast differentiation of *Fam102a*$^{+/+}$ and *Fam102a*$^{\Delta/\Delta}$ calvarial cells shown by ALP staining at 7 days of culture. Four independent experiments were performed. **d,** The effect of retroviral overexpression of Runx2 on *Alpl* mRNA expression in *Fam102a*$^{+/+}$ and *Fam102a*$^{\Delta/\Delta}$ calvarial cells (n = 4; independent experimental replicates). Data are shown as the mean ± SEM. Statistical analyses were performed using or unpaired two-sided Student' s t test (**b**) or two-way ANOVA and Sidak' s *post hoc* test (**d**). Source data are provided as a Source Data file.

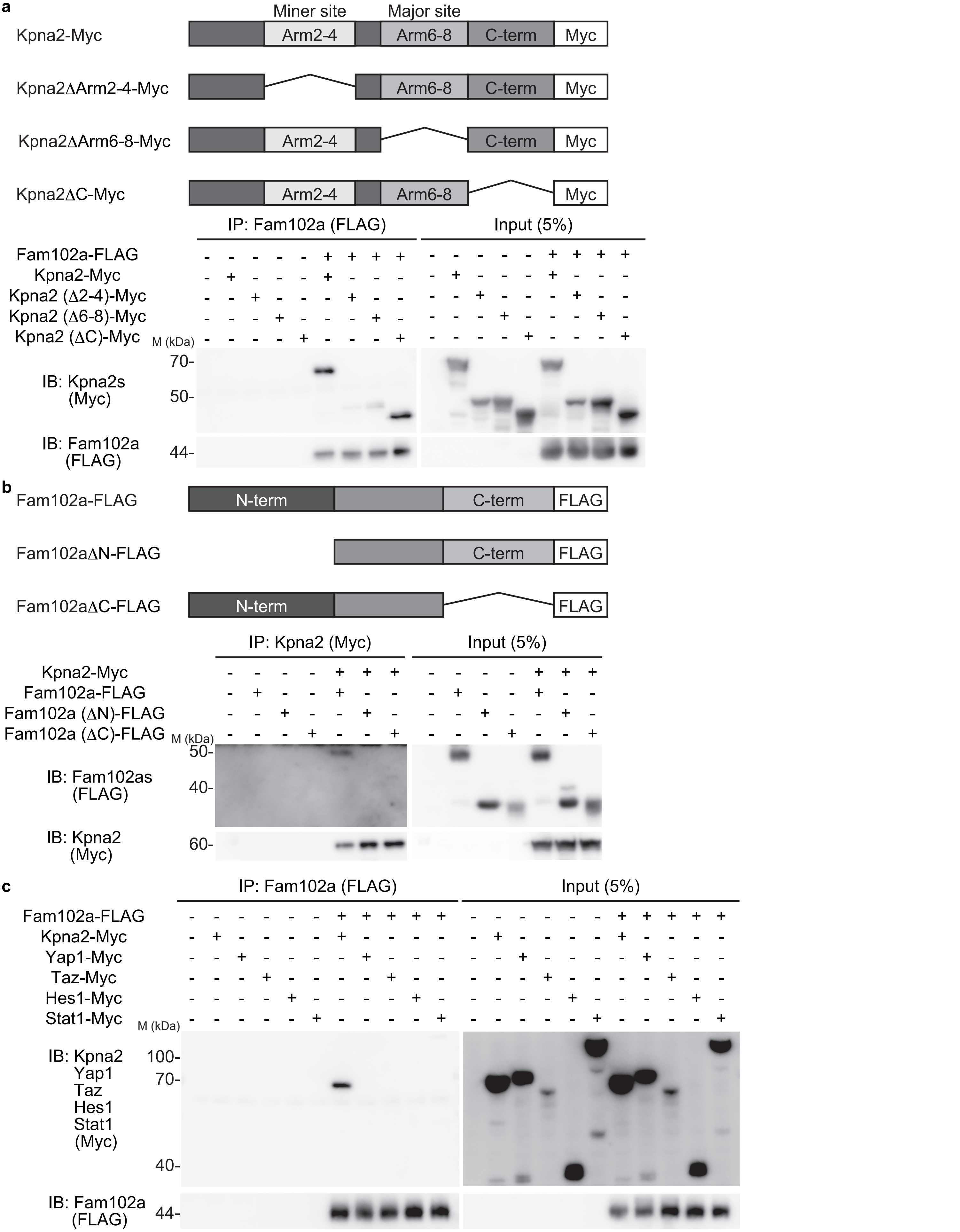

Supplementary Fig. 13: The interaction between Fam102a and Kpna2.

a, The analysis of interaction between Fam102a and Kpna2 mutants (full length, ΔArm2-4, ΔArm6-8, ΔC) examined by Co-IP assay. Three independent experiments were performed. Arm, Armadillo repeat. **b,** The analysis of interaction between Fam102a mutants (full length, ΔN, ΔC) and Kpna2 examined by Co-IP assay. Three independent experiments were performed. **c,** The analysis of the interaction between Fam102a and Runx2 binding partners examined by Co-IP assay. Three independent experiments were performed. M, molecular mass. Source data are provided as a Source Data file.

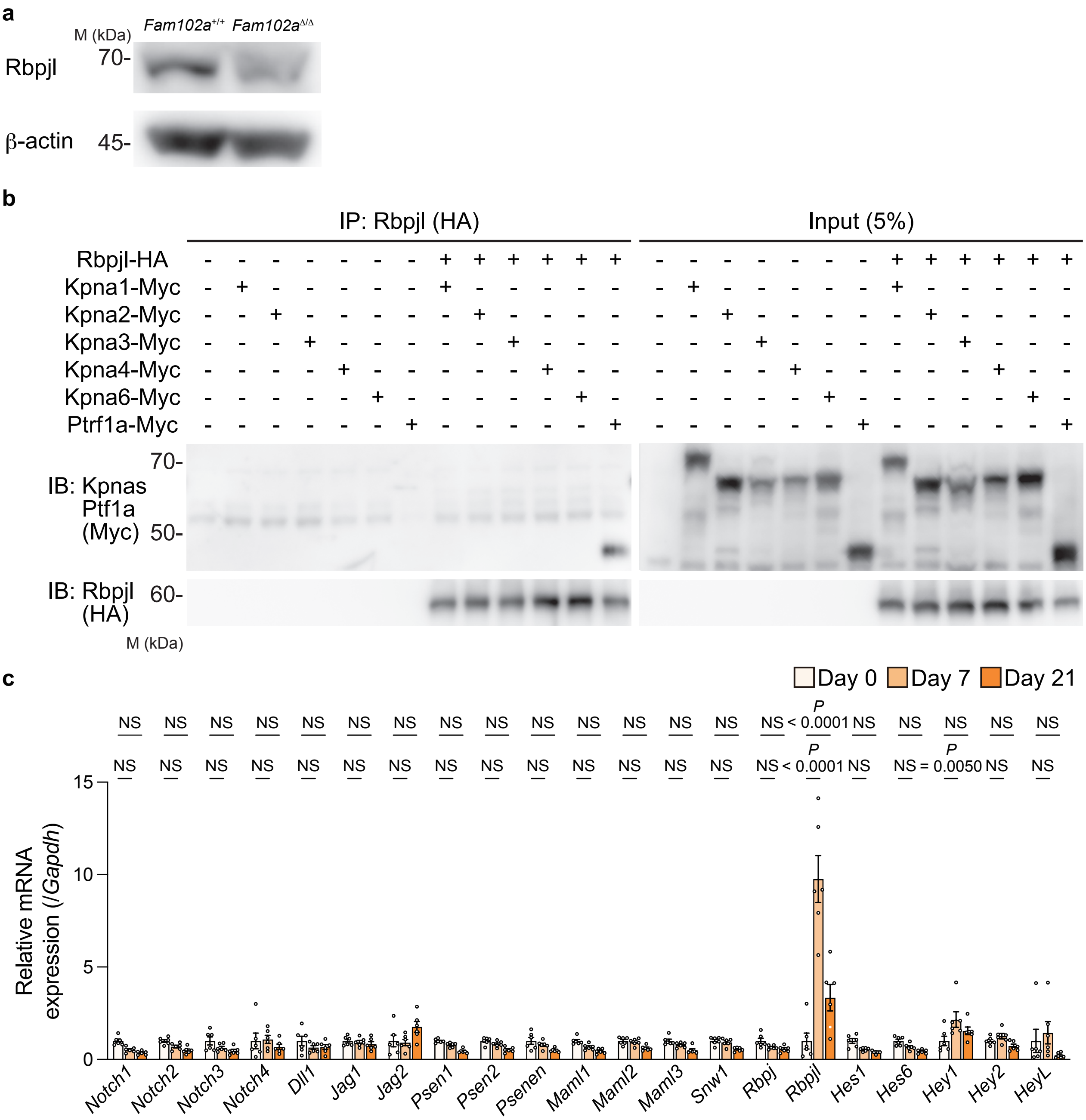

Supplementary Fig. 14: Rbpjl expression is strongly upregulated in osteoblast differentiation under the control of Fam102a.

a, Rbpjl protein expression in *Fam102a*$^{+/+}$ and *Fam102a*$^{\Delta/\Delta}$ calvarial cells cultured for 7 days. Three independent experiments were performed. **b,** The analysis of the interaction between Rbpjl, Kpna family members and Ptf1a examined by Co-IP assay. Three independent experiments were performed. **c,** mRNA expression of Notch family members and their pathway genes during osteoblastic differentiation of primary wild-type calvarial cells (n = 6; independent experimental replicates). M, molecular mass. Data are shown as the mean ± SEM. Statistical analyses were performed using two-way ANOVA and Tukey' s *post hoc* test. Source data are provided as a Source Data file.

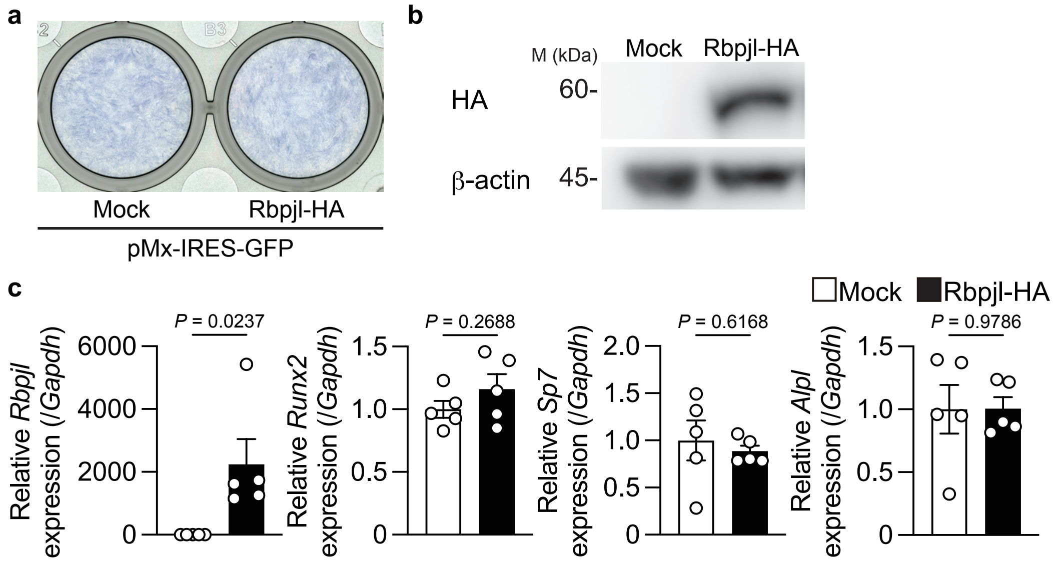

Supplementary Fig. 15: Rbpjl overexpression had no effect on osteoblast differentiation.

a–c, The effect of retroviral overexpression of Rbpjl-HA on ALP staining (a), Rbpjl protein expression (b), and osteoblastic gene expression (c) in MC3T3-E1 cells cultured in osteogenic medium for 7 days (*n* = 5; independent experimental replicates). M, molecular mass. Data are shown as the mean ± SEM. Statistical analyses were performed using unpaired two-sided Student' s *t* test. Source data are provided as a Source Data file.

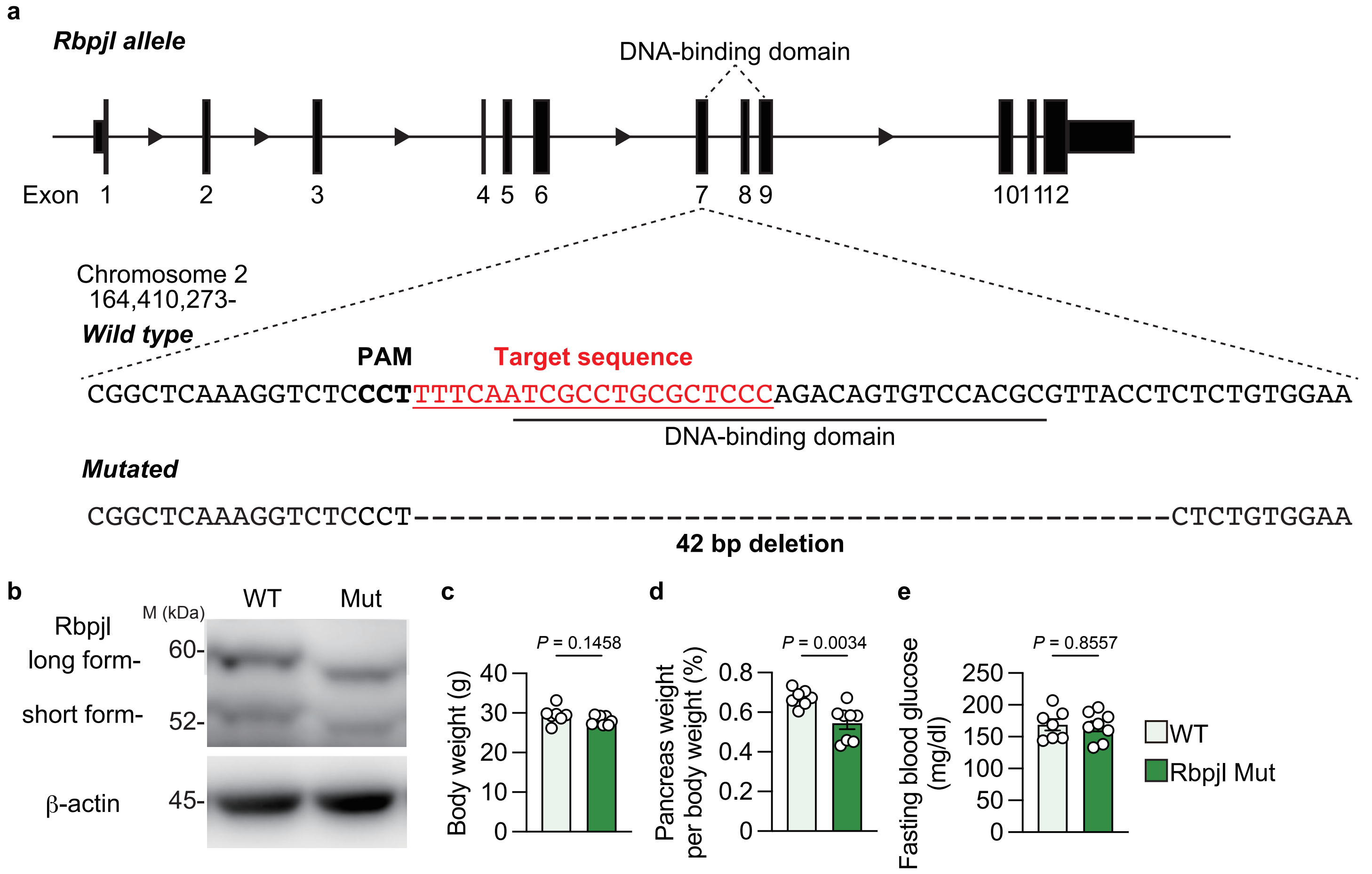

Supplementary Fig. 16: Generation of *Rbpjl*-mutated mice.

a, Targeting strategy to generate *Rbpjl*-mutated mice. Schematic diagram of the Rbpjl exon 7 sgRNA, which contained a DNA-binding domain. The target sequence is underlined in red. **b,** Rbpjl protein expression in wild-type and *Rbpjl*-mutated calvarial cells cultured for 7 days. Four independent experiments were performed. **c–e,** The effects of Rbpjl mutation on body weight (c, n = 7), pancreas weight per body weight (d, n = 7) and fasting blood glucose (e, n = 7). M, molecular mass. WT, wild-type. Mut, mutated. Data are shown as the mean ± SEM. Statistical analyses were performed using unpaired two-sided Student' s *t* test. Source data are provided as a Source Data file.

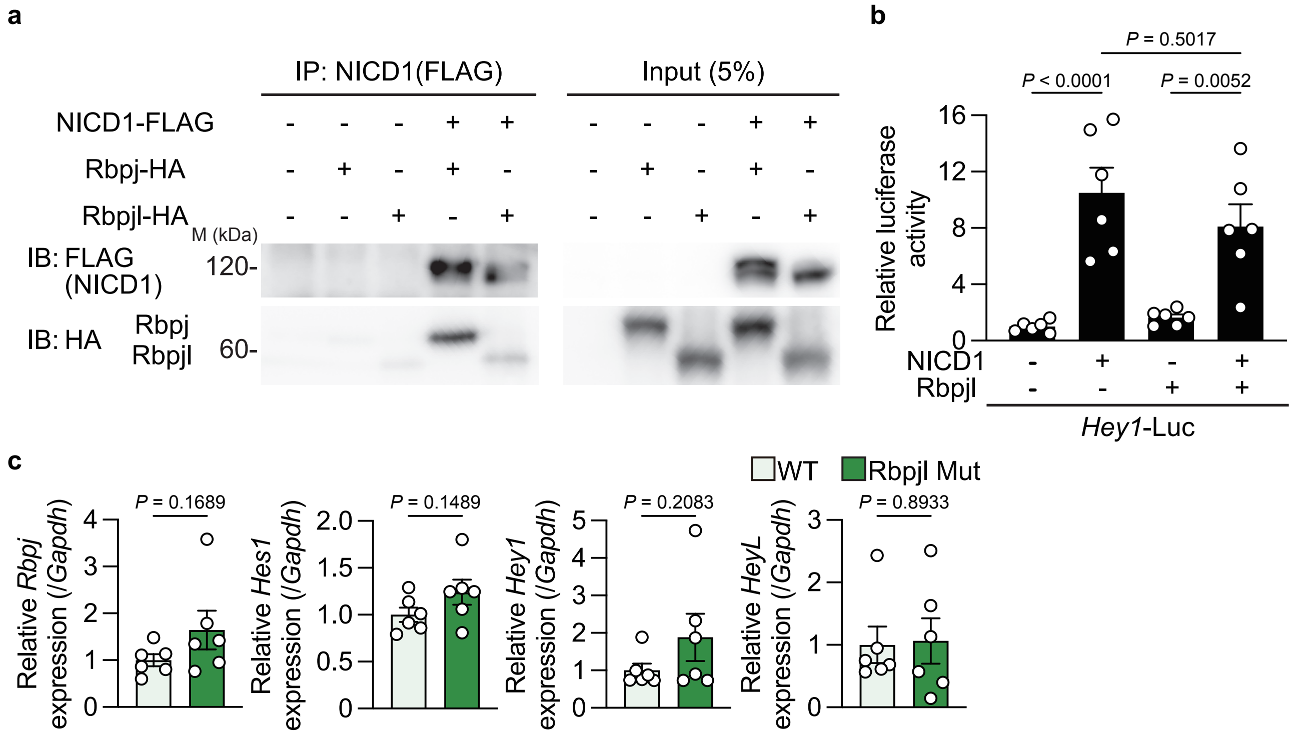

Supplementary Fig. 17: Rbpjl regulates osteoblast differentiation independently of Notch signaling.

a, The analysis of the interaction between NICD1, Rbpj, and Rbpjl examined by Co-IP assay. Three independent experiments were performed. **b,** The effect of the expression of NICD1 and Rbpjl on the reporter activity of *Hey1*-Luc in NIH3T3 cells (n = 5; independent experimental replicates). **c,** mRNA expression of *Rbpj*, and the Notch target genes *Hes1*, *Hey1*, and *HeyL* in wild-type and *Rbpjl*-mutated calvarial cells cultured in osteogenic medium for 7 days (n = 6; independent experimental replicates) (WT, wild-type; Mut, mutated). M, molecular mass. . Data are shown as the mean ± SEM. Statistical analyses were performed using one-way ANOVA and Tukey’ s *post hoc* test (**b**) or unpaired two-sided Student’ s t test (**c**). Source data are provided as a Source Data file.

a

Chromosome 2, 164,410,101-

Wild type

```
101 ATCAAGACCC CAGGCAAAAG GCTATGAAAT GCAGATAACC ATTGAGTCTA 150

151 GGCCAGTGGG CATATTAGCC AGGCTGTTAC ATGGGTCAGA AGATGAGCTG 200

201 AGGCATCTGA GAGACCACAT CTGAGAGGCC TGGGTACTGA TTCCACGTTC 250
                Exon 7                        PAM    Target sequence
251 CCCTCGCAGT ATGCATCTCC TCCGGCTCAA AGGTCTCCCT TTTCAATCGC 300

301 CTGCGCTCCC AGACAGTGTC CACGCGTTAC CTCTCTGTGG AAGATGGGGC 350
         DNA-binding domain
```

Mutated 1

```
101 ATCAAGACCC CAGGCAAAAG GCTATGAAAT GCAGATAACC ATTGAGTCTA 150

151 GGCCAGTGGG CATATTAGCC AGGCTGTTAC ATGGGTCAGA AGATGAGCTG 200

201 AGGCATCTGA GAGACCACAT CTGAGAGGCC TGGGTACTGA TTCCACGTTC 250
                Exon 7
251 CCCTCGCAGT ATGCATCTCC TCCGGCACAT CCACAAGAAT CCAAAGCGGC 300
          STOP
301 TACCAGTAAC TGCCTGATGG GAAAAGATTG AAAACCAGTA AATTTGGTCA 350

351 CAAACCAGTA AA-------- ---------C AATCGCCTGC GCTCCCAGAC 400
                                                DNA-binding
401 AGTGTCCACG -------CGT TACCTCTCTG TGGAAGATGG GGC        450
     domain
```

24 bp deletion and 86 bp insert (conclude stop codon)

Mutated 2

```
101 ATCAAGACCC CAGGCAAAAG GCTATGAAAT GCAGATAACC ATTGAGTCTA 150

151 GGCC------ ---------- ---------- ---------- ---------- 200

201 ---------- ---------- ---------- ---------- ---------- 250
                Exon 7
251 ---------- ---------- ---------- ---------- ---------- 300

301 -TGCGCTCCC AGACAGTGTC CACGCGTTAC CTCTCTGTGG AAGATGGGGC 350
         DNA-binding domain
```

147 bp deletion (exon junction defect)

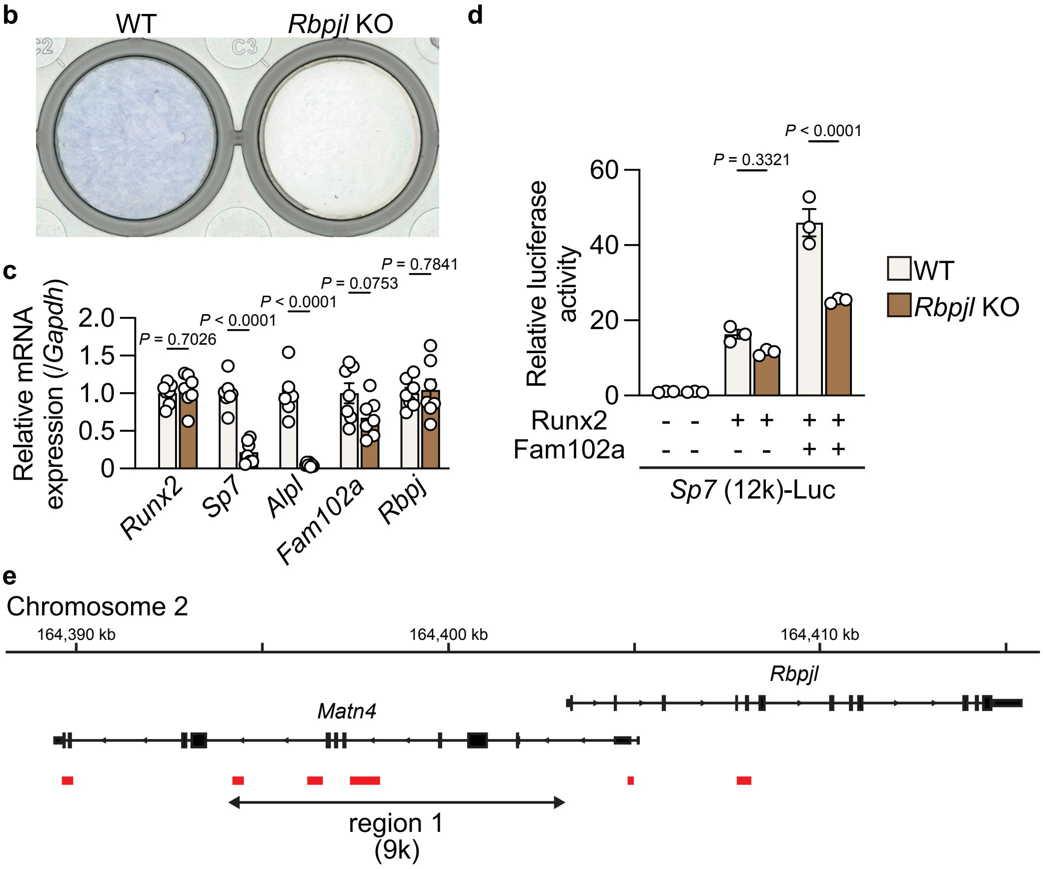

Supplementary Fig. 18: Generation of *Rbpjl* knockout osteoblastic cells.

a, Targeting strategy to generate *Rbpjl* knockout MC3T3-E1 cells. The target sequence is shown in red. The inserted bases are indicated in blue (-: deleted bases). **b,** *In vitro* osteoblastic differentiation of wild-type and *Rbpjl* knockout MC3T3-E1 cells shown by ALP staining in osteogenic medium for 7 days. **c,** mRNA expression of osteoblastic genes during differentiation in wild-type and *Rbpjl* knockout MC3T3-E1 cells (*n* = 7; independent experimental replicates). **d,** The effect of Runx2 and Fam102a expression on the reporter activity of *Sp7*-Luc (region 2) in control and Rbpjl–deficient MC3T3-E1 cells (n = 3; independent experimental replicates). **e,** Schematic view of Runx2-binding sites (red bars) in mouse *Rbpjl* promotor and enhancer region visualized using ChIP-Atlas. WT, wild-type. KO, knockout. Data are shown as the mean ± SEM. Statistical analyses were performed using unpaired two-sided Student' s *t* test. Source data are provided as a Source Data file.

Organs	*Fam102a* $^{+/+}$	*Fam102a* $^{\Delta/\Delta}$	*P* value
Brain (%)	1.770 ± 0.095	1.773 ± 0.072	0.981
Thymus (%)	0.139 ± 0.019	0.143 ± 0.004	0.854
Heart (%)	0.501 ± 0.053	0.476 ± 0.008	0.659
Lung (%)	0.564 ± 0.018	0.741 ± 0.028	0.006
Liver (%)	5.136 ± 0.106	4.710 ± 0.363	0.324
Pancreas (%)	0.762 ± 0.015	0.657 ± 0.022	0.017
Spleen (%)	0.241 ± 0.010	0.272 ± 0.007	0.053
Stomach (%)	0.620 ± 0.032	0.581 ± 0.012	0.329
Kidney (%)	0.619 ± 0.039	0.560 ± 0.007	0.211
Adrenal gland (%)	0.012 ± 0.001	0.012 ± 0.000	0.564
Subcutaneous adipose (%)	0.569 ± 0.100	0.620 ± 0.038	0.662
Gastrocnemius muscle (%)	0.666 ± 0.022	0.679 ± 0.008	0.591
Soleus muscle (%)	0.040 ± 0.002	0.045 ± 0.002	0.161
Body weight (g)	26.3 ± 1.7	24.3 ± 0.7	0.332
Fasting blood glucose (mg/dl)	125 ± 16.0	124 ± 16.2	0.967

Supplementary Table 1: List of organ weights in *Fam102a*$^{\Delta/\Delta}$ mice.

Data are shown as the mean ± SEM. Statistical analyses were performed using unpaired two-sided Student's *t* test.

Target gene	Orientation	Sequence 5'→3'
Gapdh	Forward	AACTTTGGCATTGTGGAAGG
Gapdh	Reverse	GGATGCAGGGATGATGTTCT
Fam102a	Forward	TGAACGGGGTCCTCTTTTGC
Fam102a	Reverse	CTCTTCCGCCATCGTACACAG
Runx2	Forward	AACGATCTGAGATTTGTGGGC
Runx2	Reverse	CCTGCGTGGGATTTCTTGGTT
Sp7	Forward	ATGGCGTCCTCTCTGCTTG
Sp7	Reverse	TGAAAGGTCAGCGTATGGCTT
Alpl	Forward	CCAACTCTTTTGTGCCAGAGA
Alpl	Reverse	GGCTACATTGGTGTTGAGCTTTT
Bglap	Forward	CTGACCTCACAGATGCCAAGC
Bglap	Reverse	TGGTCTGATAGCTCGTCACAAG
Rbpjl	Forward	ACTCCGGTGCCTCTCATCAG
Rbpjl	Reverse	CTACGCACACCAAGGAACGA
Col1a1	Forward	GCTCCTCTTAGGGGCCACT
Col1a1	Reverse	CCACGTCTCACCATTGGGG
Fos	Forward	CGGGTTTCAACGCCGACTA
Fos	Reverse	TTGGCACTAGAGACGGACAGA
Nfatc1	Forward	GCCTTTTGCGAGCAGTATCTG
Nfatc1	Reverse	GCTGCACCTCGATCCGAAG
Notch1	Forward	GATGGCCTCAATGGGTACAAG
Notch1	Reverse	TCGTTGTTGTTGATGTCACAGT
Notch2	Forward	CAGGAGGTGATAGGCTCTAAG
Notch2	Reverse	GAAGCACTGGTCTGAATCTTG
Notch3	Forward	TGCCAGAGTTCAGTGGTGG
Notch3	Reverse	CACAGGCAAATCGGCCATC
Notch4	Forward	GGAGATGTGGATGAGTGTCTGG
Notch4	Reverse	TGGCTCTGACAGAGGTCCATCT
Dll1	Forward	CCCATCCGATTCCCCTTCG
Dll1	Reverse	GGTTTTCTGTTGCGAGGTCATC
Jag1	Forward	AATCGCATCGTACTGCCTTTC

Jag1	Reverse	GTGTCATTACTGGAATCCCAGG
Jag2	Forward	CGCTGCTATGACCTGGTCAATG
Jag2	Reverse	TGTAGGCGTCACACTGGAACTC
Psen1	Forward	ATACCTGCACCTTTGTCCTACT
Psen1	Reverse	GCTCAGGGTTGTCAAGTCTCT
Psen2	Forward	GAAGACTCCTACGACAGTTTTGG
Psen2	Reverse	CACCAGGACGCTGTAGAAGAT
Psenen	Forward	ATGAACTTGGAGCGGGTATCC
Psenen	Reverse	CGAGGAACGCCTCTCTGAAG
Maml1	Forward	AACATGCCCTTCCGGTCATT
Maml1	Reverse	CTGGCCACAGACACAGTAGG
Maml2	Forward	CTGCTCAGTATTCGGGTGGC
Maml2	Reverse	CCCATGAGACGTAGACATGAGG
Maml3	Forward	AACAGCGGAACCCATACCC
Maml3	Reverse	CCTGGTTTCTCACAGCTGCTA
Snw1	Forward	CGGCAGGGACAGTCCAAAG
Snw1	Reverse	ACCTTCTGCGACACAGATTTC
Rbpj	Forward	GCGGATAAAGGTCATCTCCA
Rbpj	Reverse	TTCCTGAAGCAATGCACAAG
Hes1	Forward	AAGATAGCTCCCGGCATTCC
Hes1	Reverse	ACCTCGTTCATGCACTCGCT
Hes6	Forward	CAACGAGAGTCTTCAGGAGCTGCG
Hes6	Reverse	GCATGCACTGGATGTAGCCAGCAG
Hey1	Forward	CATGAAGAGAGCTCACCCAGA
Hey1	Reverse	CGCCGAACTCAAGTTTCC
Hey2	Forward	GAGAACAATTACCCTGGGCAC
Hey2	Reverse	GGTAGTTGTCGGTGAATTGGAC
Heyl	Forward	AGATGCAAGCCCGGAAGAA
Heyl	Reverse	CGCAATTCAGAAAGGCTACTGTT

Supplementary Table 2: Primer pairs for quantitative PCR

Gene	Target	Orientation	Sequence 5'→3'
Sp7	Site 1	Forward	CTGACAACTTGCCCATATTCC
	Site 1	Reverse	CTCCCCAAAGAAGGGATCTG
	Site 2	Forward	CTCCGAGTCAAGAGTAGGATTGT
	Site 2	Reverse	CTCTCTCAGGCCAGCTCACT
	Site 3	Forward	TATGCTCTGCAAACCACAGC
	Site 3	Reverse	AGAGGCAGCCTGGAGAAAGT
Rbpjl	Site	Forward	TGCTGGGTCTGGCTTCTACTGC
	Site	Reverse	TTTGCTCTGGGCAGGGACAC

Supplementary Table 3: Primer pairs for Cut & Run
