# Supplementary material for: Fam102a translocates Runx2 and Rbpjl to facilitate Osterix expression and bone formation
Source: Nat Commun. 2025 Jan 2;16:9. doi: 10.1038/s41467-024-55451-z (PMC11695619; doi:10.1038/s41467-024-55451-z)
Supplement: Supplementary file 2 — Reporting summary [file 41467_2024_55451_MOESM2_ESM.pdf]

nature portfolio

Corresponding author(s): Mikihito Hayashi

Last updated by author(s): Nov 22, 2024

Reporting Summary

Nature Portfolio wishes to improve the reproducibility of the work that we publish. This form provides structure for consistency and transparency in reporting. For further information on Nature Portfolio policies, see our Editorial Policies and the Editorial Policy Checklist.

Statistics

For all statistical analyses, confirm that the following items are present in the figure legend, table legend, main text, or Methods section.

n/a	Confirmed	
☐	☒	The exact sample size (*n*) for each experimental group/condition, given as a discrete number and unit of measurement
☐	☒	A statement on whether measurements were taken from distinct samples or whether the same sample was measured repeatedly
☐	☒	The statistical test(s) used AND whether they are one- or two-sided<br>*Only common tests should be described solely by name; describe more complex techniques in the Methods section.*
☒	☐	A description of all covariates tested
☐	☒	A description of any assumptions or corrections, such as tests of normality and adjustment for multiple comparisons
☐	☒	A full description of the statistical parameters including central tendency (e.g. means) or other basic estimates (e.g. regression coefficient) AND variation (e.g. standard deviation) or associated estimates of uncertainty (e.g. confidence intervals)
☐	☒	For null hypothesis testing, the test statistic (e.g. *F*, *t*, *r*) with confidence intervals, effect sizes, degrees of freedom and *P* value noted<br>*Give P values as exact values whenever suitable.*
☒	☐	For Bayesian analysis, information on the choice of priors and Markov chain Monte Carlo settings
☒	☐	For hierarchical and complex designs, identification of the appropriate level for tests and full reporting of outcomes
☒	☐	Estimates of effect sizes (e.g. Cohen's *d*, Pearson's *r*), indicating how they were calculated

Our web collection on statistics for biologists contains articles on many of the points above.

Software and code

Policy information about availability of computer code

Data collection	BZ-X_Analyzer v1.4.0.1 (Keyence), Bio-Rad CDX Manager v3.11517.0823 (Bio-Rad), CLC Genomics workbench v12.0.3(CLC bio).
Data analysis	Graph Pad Prism v8 (Graph Pad Prism Software), ImageJ v1.53k (National Institutes of Health), BON-FCS v10.01.37.47-H-64 (RATOC).

For manuscripts utilizing custom algorithms or software that are central to the research but not yet described in published literature, software must be made available to editors and reviewers. We strongly encourage code deposition in a community repository (e.g. GitHub). See the Nature Portfolio guidelines for submitting code & software for further information.

Data

Policy information about availability of data

All manuscripts must include a data availability statement. This statement should provide the following information, where applicable:

- Accession codes, unique identifiers, or web links for publicly available datasets
- A description of any restrictions on data availability
- For clinical datasets or third party data, please ensure that the statement adheres to our policy

The RNAseq data produced in this study were deposited to the public data base (GSE211533: https://www.ncbi.nlm.nih.gov/geo/query/acc.cgi?acc=GSE211533). Source data are provided in our paper. All other data supporting the findings of this study are available from the corresponding author upon reasonable request, as these data are useed in our future studies.

Research involving human participants, their data, or biological material

Policy information about studies with human participants or human data. See also policy information about sex, gender (identity/presentation), and sexual orientation and race, ethnicity and racism.

Reporting on sex and gender	Human research participants is not relevant to this study.
Reporting on race, ethnicity, or other socially relevant groupings	Same as the above.
Population characteristics	Same as the above.
Recruitment	Same as the above.
Ethics oversight	Same as the above.

Note that full information on the approval of the study protocol must also be provided in the manuscript.

Field-specific reporting

Please select the one below that is the best fit for your research. If you are not sure, read the appropriate sections before making your selection.

☒ Life sciences ☐ Behavioural & social sciences ☐ Ecological, evolutionary & environmental sciences

For a reference copy of the document with all sections, see nature.com/documents/nr-reporting-summary-flat.pdf

Life sciences study design

All studies must disclose on these points even when the disclosure is negative.

Sample size	No sample-size calculation was performed. The sample sizes were guided by previous studies using similar studies (Liu, A et al., Nature Communications, 2024, Hayashi, M et al., Nature, 2012)
Data exclusions	No data were excluded from the analyses.
Replication	To confirm the reproducibility of the experimental results, we performed at least three independent biological replicates. The number of biological replicates (n) was reported for each experiment.
Randomization	The order of bone analyses for different genotypes was randomized. The order of bone analyses for different genotypes was randomized. Animals of both sexes were analyzed independently, and all samples were prepared and treated identically to eliminate covariate effects. For image analyses, random fields of view were selected. Other data were based on quantitative measurements that cannot be influenced by experimenter bias.
Blinding	Blinding was used for bone histomorphometric and micro-CT analyses. All other datasets are based on objectively measurable data; thus, blinding does not affect these values.

Reporting for specific materials, systems and methods

We require information from authors about some types of materials, experimental systems and methods used in many studies. Here, indicate whether each material, system or method listed is relevant to your study. If you are not sure if a list item applies to your research, read the appropriate section before selecting a response.

Materials & experimental systems

n/a	Involved in the study	
☐	☒	Antibodies
☐	☒	Eukaryotic cell lines
☒	☐	Palaeontology and archaeology
☐	☒	Animals and other organisms
☒	☐	Clinical data
☒	☐	Dual use research of concern
☒	☐	Plants

Methods

n/a	Involved in the study	
☒	☐	ChIP-seq
☒	☐	Flow cytometry
☒	☐	MRI-based neuroimaging

Antibodies

Antibodies used	Anti-HA-tag (TANA2, MBL, Cat# M180-3, 1:1000 dilution for western blotting)<br>Anti-Myc-tag (My3, MBL, Cat# M192-3, 1:10000 dilution for western blotting)<br>Anti-LaminB1 (A-11, Santa Cruz, Cat# sc-377000, 1:500 dilution for western blotting)<br>Anti-α-tubulin (DM1A, Santa Cruz, Cat# sc-32293, 1:500 dilution for western blotting)<br>Anti-β-actin (AC-15, Sigma-Aldrich, Cat# A5441, 1:1000 dilution for western blotting)<br>Anti-FLAG® M2 (M2, Sigma-Aldrich, Cat# F1804, 1:1000 dilution for western blotting, 1:50 dilution for CUT&RUN)<br>Anti-Runx2 (D1L7F, Cell Signaling Technology, Cat# 12556, 1:1000 dilution for western blotting, 1:250 dilution for immunocytochemical staining)<br>Anti-Phospho-ERK (D13.14.4E, Cell Signaling Technology, Cat# 4370, 1:1000 dilution for western blotting)<br>Anti-ERK (137F5, Cell Signaling Technology, Cat# 4695, 1:1000 dilution for western blotting)<br>Anti-Phospho-p38 (D3F9, Cell Signaling Technology, Cat# 4511, 1:1000 dilution for western blotting)<br>Anti-p38 (D13E1, Cell Signaling Technology, Cat# 8690, 1:1000 dilution for western blotting)<br>Anti-Phospho-JNK (81E11, Cell Signaling Technology, Cat# 4668, 1:1000 dilution for western blotting)<br>Anti-JNK (N/A, Cell Signaling Technology, Cat# 9252, 1:1000 dilution for western blotting)<br>Anti-Rbpjl (prepared by Sigma-Aldrich against a sequence near the C terminus, 1:250 dilution for western blotting, 1:250 dilution for immunocytochemical staining)<br>Anti-mouse IgG (N/A, Santa Cruz, Cat# sc-2025, 1:10000 dilution for western blotting)<br>Anti-rabbit IgG (N/A, Santa Cruz, Cat# sc-2027, 1:10000 dilution for western blotting)<br>Anti-DDDDK-tag Magnetic Beads (FLA-1GS, MBL, Cat# M185-11, 1:5 dilution for co-immunoprecipitation)<br>Anti-HA-tag Magnetic Beads (TANA2, MBL, Cat# M180-11, 1:5 dilution for co-immunoprecipitation)<br>Anti-Myc-tag Magnetic Beads (PL14, MBL, Cat# M047-11, 1:5 dilution for co-immunoprecipitation)<br>Alexa Fluor® 488-labeled Donkey anti-Rabbit IgG (N/A, Invitrogen, Cat# A21206, 1:1000 dilution for immunocytochemical staining)<br>Hoechst® 33342 (N/A, Invitrogen, Cat# H1399, 1:10000 dilution for immunocytochemical staining)<br>Anti-HA-tag (C29F4, Cell Signaling Technology, Cat# 3724, 1:1000 dilution for western blotting, 1:50 dilution for CUT&RUN)DA1E<br>Anti-rabbit IgG (DA1E, Cell Signaling Technology, Cat# 66362, 1:20 dilution for CUT&RUN)
Validation	Antibodies without anti-Rbpjl used here are commercially available. The rabbit anti-Rbpjl antibody was prepared by Sigma-Aldrich against a sequence near the C terminus of mouse Rbpjl, and affinity purified using the synthetic peptide. Antibodies for western blotting, immunocytochemical staining and co-immunoprecipitation were purchased from MBL, Santa cruz, Sigma-Aldrich, Cell Signaling Technology and Invitrogen. These antibodies are quality control tested for western blotting as mentioned in manufacturer's website.<br>Anti-HA-tag (MBL); https://ruo.mbl.co.jp/bio/dtl/A/?pcd=M180-3<br>Anti-Myc-tag; https://ruo.mbl.co.jp/bio/dtl/A/index.html?pcd=M192-3<br>Anti-LaminB1; https://www.scbt.com/ja/p/lamin-b1-antibody-a-11<br>Anti-α-tubulin; https://www.scbt.com/ja/p/alpha-tubulin-antibody-dm1a<br>Anti-β-actin; https://www.sigmaaldrich.com/JP/ja/product/sigma/a5441<br>Anti-FLAG® M2; https://www.sigmaaldrich.com/JP/ja/product/sigma/f1804<br>Anti-Runx2; https://www.cellsignal.jp/products/primary-antibodies/runx2-d1l7f-rabbit-mab/12556<br>Anti-Phospho-ERK; https://www.cellsignal.jp/products/primary-antibodies/phospho-p44-42-mapk-erk1-2-thr202-tyr204-d13-14-4e-xp-rabbit-mab/4370<br>Anti-ERK ; https://www.cellsignal.jp/products/primary-antibodies/p44-42-mapk-erk1-2-137f5-rabbit-mab/4695<br>Anti-Phospho-p38; https://www.cellsignal.jp/products/primary-antibodies/phospho-p38-mapk-thr180-tyr182-d3f9-xp-rabbit-mab/4511<br>Anti-p38; https://www.cellsignal.jp/products/primary-antibodies/p38-mapk-d13e1-xp-rabbit-mab/8690<br>Anti-Phospho-JNK; https://www.cellsignal.jp/products/primary-antibodies/phospho-sapk-jnk-thr183-tyr185-81e11-rabbit-mab/4668<br>Anti-JNK; https://www.cellsignal.jp/products/primary-antibodies/sapk-jnk-antibody/9252<br>Anti-mouse IgG; https://www.scbt.com/ja/p/normal-mouse-igg<br>Anti-rabbit IgG; https://datasheets.scbt.com/sc-2027.pdf<br>Anti-DDDDK-tag Magnetic Beads; https://ruo.mbl.co.jp/bio/dtl/A/index.html?pcd=M185-11R<br>Anti-HA-tag Magnetic Beads; https://ruo.mbl.co.jp/bio/dtl/A/?pcd=M180-11<br>Anti-Myc-tag Magnetic Beads; https://ruo.mbl.co.jp/bio/dtl/A/?pcd=M047-11<br>Alexa Fluor® 488-labeled Donkey anti-Rabbit Ig; https://www.thermofisher.com/antibody/product/Donkey-anti-Rabbit-IgG-H-L-Highly-Cross-Adsorbed-Secondary-Antibody-Polyclonal/A-21206<br>Hoechst® 33342; https://www.thermofisher.com/order/catalog/product/H1399<br>Anti-HA-tag (Cell Signaling Technology); https://www.cellsignal.jp/products/primary-antibodies/ha-tag-c29f4-rabbit-mab/3724<br>Anti-rabbit IgG (Cell Signaling Technology); https://www.cellsignal.jp/products/primary-antibodies/rabbit-da1e-mab-igg-xp-isotype-control-cut-run/66362

Eukaryotic cell lines

Policy information about cell lines and Sex and Gender in Research

Cell line source(s)	MC3T3-E1 (RBRC-RCB1126, Lot. 057), HEK293 (RBRC-RCB1637, Lot. 024), HEK293T(RBRC-RCB2202, Lot. 021), NIH3T3 cells (RBRC-RCB1862, Lot. 011) obtained from Riken BRC.
Authentication	The cells were originally authenticated by ATCC; no further authentication was performed during this study.
Mycoplasma contamination	Not tested
Commonly misidentified lines (See ICLAC register)	No commonly misidentified lines

Animals and other research organisms

Policy information about studies involving animals; ARRIVE guidelines recommended for reporting animal research, and Sex and Gender in Research

Laboratory animals	Ctsk-Cre mice were kindly provided by Dr. T. Nakamura (Nakamura, T et al., Cell 2007). Nfatc1-floxed mice were kindly provided by Dr. A. Rao (Martinez, GJ et al., Immunity 2015). Actb-Cre and Sp7-Cre mice were generated as previously described (Nakashima, T et al., Nat Med 2011; Rodda, SJ et al., Development 2006). Fam102a-floxed mice were generated as follows a targeting vector was designed to insert a single loxP site upstream of exon 2, and a loxP-flanked neomycin resistance cassette downstream of exon 3 of the Fam102a gene. Rbpjl-mutated mice were generated by i-GONAD method. Wild-type mice (C57BL/6J) were obtained from CLEA Japan. All of the mice were maintained under specific pathogen-free conditions. Mice were maintained in a temperature and humidity-controlled room on a 12-hour light cycle with ad libitum access to water and a standard laboratory chow diet.
Wild animals	The study did not involve wild animals.
Reporting on sex	Considering that Fam102a expression could be induced by estrogen stimulation in mice (Wang, DY et al., Molecular Endocrinology 2004), male mice were chosen for most of the experiments.
Field-collected samples	The study did not involve samples collected from the field.
Ethics oversight	All experiments were performed with the approval of the Institutional Animal Care and Use Committee of Institute of Science Tokyo.

Note that full information on the approval of the study protocol must also be provided in the manuscript.

Plants

Seed stocks	The study did not involve plants.
Novel plant genotypes	Same as the above.
Authentication	Same as the above.
